# The Effect of Tropical Environment on Fatigue Failure in Royal Malaysian Airforce (RMAF) Aircraft Structure and Operational Readiness

**DOI:** 10.3390/ma14092414

**Published:** 2021-05-06

**Authors:** Arvinthan Venugopal, Roslina Mohammad, Md Fuad Shah Koslan, Syed Roslee Sayd Bakar, Alizarin Ali

**Affiliations:** 1 RMAF Centre of Aerospace Engineering Services Establishment, Subang Airbase, Shah Alam 40000, Malaysia; arvinthanvenugopal@yahoo.com (A.V.); md.fuad@airforce.mil.my (M.F.S.K.); 2 Razak Faculty of Technology and Informatics, Universiti Technology Malaysia, Jalan Sultan Yahya Petra, Kuala Lumpur 54100, Malaysia; 3 Science & Technology Research Institute for Defence (STRIDE) Kajang, Selangor 43000, Malaysia; syroslee.sybakar@stride.gov.my; 4 CAIDMARK Sdn Bhd, Damansara Utama Petaling Jaya, Selangor 47400, Malaysia; alizarin@caidmark.com.my

**Keywords:** longeron, corrosion, fatigue, aluminum 2024, aircraft structure integrity program

## Abstract

The environmental condition in which the Royal Malaysian Airforce is currently operating its aircraft is prone to corrosion. This is due to the high relative humidity and temperature. With most of its aircraft being in the legacy aircraft era, the aircraft’s main construction consists of the aluminium 2024 material. However, this material is prone to corrosion, thus reducing fatigue life and leading to fatigue failure. Using the concept of either Safe Life or Damage Tolerance as its fatigue design philosophy, the RMAF adopts the Aircraft Structure Integrity Program (ASIP) to monitor its structural integrity. With the current problem of not having the structural limitation on corrosion-damaged structure, the RMAF has embarked on its fatigue testing method. Finite Element (FE) studies and flight tests were conducted, and the outcome is summarized. The conclusion is that the longeron tested on the aircraft can withstand the operational load, and its yield strength is below the ultimate yield strength of the material. These research outcomes will also enhance the ASIP for other aircraft platforms in the RMAF fleet for its structure life assessment or service life extension program.

## 1. Introduction

The current environmental condition around the world has a significant influence on the operating condition of the aircraft. Its most significant influence is on the structural condition of the aircraft. The highest and most common issues affecting the aircraft structure are corrosion, which results from metal and nature interaction. The leading agent contributing to deterioration is mainly the environment, and this will also accelerate the corrosion process. The corrosion causes degradation of the material characteristics, worsening or weakening the particular object [1].

The surrounding condition is related to its humidity and also temperature. This plays an important role in absorbing moisture into the exposed aircraft structure [2]. Based on the Meteorological Department’s current data (Met Malaysia), the average humidity for most of the states in Malaysia is approximately 50%, with an average temperature of 27 °C, the yearly wetness (TOW) in peninsular Malaysia is around 0.783 fractional hours. Based on ISO 9223, it is classified that the corrosion level in Malaysia is at class 5, in which the corrosion rate is higher [3].

The coast and adjacent areas onshore and offshore are an essential part of local ecosystems, forming gulfs, bay, and estuaries, sometimes mixing fresh and salty waters. The infrastructure of most airbases in Malaysia constitutes defending the country’s sovereignty either through air or sea. [4] explained that the sea distance’s influence is one of the most critical aspects of Marine Atmospheric Corrosion (MAC) in coastal areas. Keeping in mind that the corrosion and atmospheric salinity are inter-related, the variation in corrosion compared with the distance of its location to the shore should be exponential.

Corrosion is the primary concern affecting metallic structure in its material characteristic and performance. For the aluminium structure, corrosion occurs through the electrochemical reaction between the metallic matrix and its reinforcement. The most common types of corrosion occurring on metallic structures are crevice corrosion, intergranular corrosion, and galvanic corrosion. Besides that, pitting corrosion occurs on high-strength aluminium alloys. Pitting corrosion is very significant in its characteristics of mass and energy reduction of the structure, which accelerates the structure’s degradation and leads towards failure subjected to a corrosive surrounding environment [5]. The effect of pitting corrosion will initiate the crack initiation and, with continuous cyclic loading, can cause fatigue failure of the structure. The pit caused by the corrosion and experiencing stress load will become a stress concentrator and cause fatigue cracking. This type of corrosion’s overall process includes pit initiation, growth, and crack initiation, plus the crack growth till failure.

### 1.1. Military Aircraft Corrosion Fatigue and Protection

Many in-service aircraft are required to operate beyond their original design life, partly due to the accelerating costs of replacement and the ability to upgrade systems in old airframes. As part of the life extension program and aging aircraft audit, RMAF has conducted the structural inspection of several military airframes [6]. This involves dismantling a representative example of older airframes of a particular aircraft type and making a thorough inspection of each component to assess its condition. This teardown of aircraft has enabled the assessment of components that would not commonly be addressed during routine maintenance because of their inaccessibility. Historically, most of the structural failures examined have been in metallic materials, reflecting the predominance of metallic structures in aircraft. The RMAF currently operates aircraft from legacy years, such as the C-130H, S61A-4, MiG-29N, and F/A-18D, which have operated for around 24 to over 50 years.

The main factor affecting the aircraft structure advancing towards this corrosion fatigue is its operating mission and parent base location. The bases in which these legacy aircraft are operating are located near the coast, which is prone to corrosion due to its salt-laden environment. The operational mission that is inducing fatigue and over the sea operation also plays an important role in structural integrity. This is an alarming issue in the fleet of legacy aircraft and other aircraft and needs to be addressed urgently to prevent catastrophic failure.

Billions of dollars in assets are lost every year by the military and industry, and just as the industry is making significant changes in the way they do business, so is the military. Many factors are now being considered when evaluating corrosion prevention. Today, the many different methods utilized range from the blockage of moisture and other atmospheric contaminants on metal with ordinary greases to silica gel to absorb moisture to expensive alternatives such as dehumidification. The protection method currently used is to address water-displacing products, water absorption, dehumidification, and vapor corrosion inhibitors [7].

Numerous improvements have been made to the aircraft in the RMAF over the years. These include using more corrosion-resistant aluminium alloys, improved finish systems, and increased use of corrosion-inhibiting sealants. Unfortunately, these improvements cannot in themselves guarantee a permanently corrosion-free airplane. A certain amount of corrosion is inevitable, even with the best of care. Furthermore, as the airplane ages, corrosion problems tend to grow. This leads to an increase in labor, parts, and material costs. There are currently two methods utilized in the aircraft Corrosion Prevention and Control Program (CPCP). The automated outdoor washing facility (Bird Wash) is used to rinse the aircraft upon return from sea operations or is performed on aircraft at a stipulated interval. Besides that, the usage of corrosion-prevention compounds on the structural material protects the surface.

### 1.2. Location of RMAF Bases

**Figure 1:**
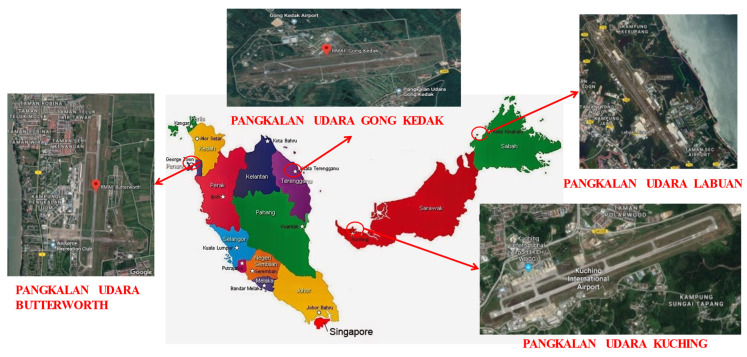
Location of RMAF Bases.

The RMAF has 13 bases in the country, which house the various administrative headquarters, flying squadron, training centers, and Research and Development (R&D) Centers. The bases in peninsular Malaysia are under the No. 1 Air Region’s command, while the bases in Sabah, Sarawak, and Labuan come under the No. 2 Air Region’s command. Malaysia being a country that is mainly surrounded by sea, it is undeniable that most of the bases are located close to the seashore area. These areas are emitting a high concentration of salt, which is a contributor to corrosion. Furthermore, the operating temperature of the areas located close to the seashore is around 30–32 °C. The bases that are located close to the shore line are depicted in Figure 1.

## 2. Problem Statement

Aluminium alloy 2024 is one of the most famous types of material used in the aviation industry as the aircraft structure material. It has some excellent characteristics such as high specific strength and excellent plasticity. However, this material is also prone to corrosion in Malaysia’s salt-laden environment due to chloride ions penetration, which will reduce fatigue life and cause failure. Fatigue failure, which is caused by continuous cyclic loading, will be accelerated due to corrosion. Aircraft structures, when built, are predicted to endure long lives with exposure to the corrosive environment. However, the fatigue resistance of the aluminium alloy 2024 reduces significantly when the material is affected by corrosion.

Most of the aircraft’s primary structure elements (PSE) are built from aluminium alloy 2024-TXX. This material is chosen due to its high-strength properties. Nevertheless, this material is subjected to corrosion such as exfoliation corrosion, stress corrosion cracking, and pitting corrosion. With many aircraft in the fleet already in the aging category, the operators are still keen to keep them in service until its Planned Withdrawal Date (PWD). It requires periodic maintenance to measure the rate of corrosion and fatigue damage on the structure. The current practice on corrosion removal is with a maximum allowable reduction of 10% of the total thickness, otherwise it must be replaced. Fatigue cracks initiate from contortion points and finally reduce the component’s fatigue life.

Hence, this will affect the airframe’s structural integrity, resulting in structural deterioration and frequent downtime for maintenance or unscheduled repair. Having frequent downtime will jeopardize the operational availability of the particular squadron or fleet. This will reduce the readiness of the RMAF in ensuring a sustainable fleet to carry out its intended mission. Besides that, it is desirable to have an analytical tool that will assess the effect of this corrosion damage on the component’s remaining fatigue life.

The RMAF Aircraft Structure Integrity Program (ASIP) is a crucial tool to manage four critical elements in ensuring the aircraft’s structural integrity is airworthy. This cradle-to-grave program monitors elements such as Usage Monitoring (UM), Condition Monitoring (CM), Fatigue Management (FM), and Environmental Degradation Management (EDM). The four elements here are interrelated to each other and provide the basis for continuous airworthiness monitoring.

## 3. Fatigue Life Management

Fatigue Life Management (FLM) involves the synthesis of usage data, data analysis methods and processes, and fatigue or damage-tolerance analysis. Analysis methods for FLM are directly related to the aircraft’s certification basis and are therefore influenced by either:

- Safe life aircraft—for aircraft designed and operated under this design philosophy, the analysis determines fatigue life expended index (FLEI) (fatigue index) (FI) for each aircraft.
- Damage Tolerance—for aircraft designed and operated under damage-tolerance philosophy, the analysis determines aircraft inspection thresholds and intervals for the fleet or individual aircraft.

FLM delivers outputs such as FLEI or FI, inspection intervals, predictions of remaining aircraft fatigue life and planned withdrawal date (PWD) assessment, and recommended fleet management actions [8].

### Miner’s Rule

The most common and effective way to determine any aircraft’s structural life is to monitor fatigue life. However, there is limited technology in the legacy-type aircraft to monitor the structural usage and condition related to fatigue damage. With the advancement of technology, the situation has changed with the latest aircraft being equipped with a flight data recorder (FDR) system and fitted with strain gauges. The parameters recorded, such as flying hours, fatigue index, and strain data, help determine the fatigue damage of the aircraft fatigue critical location (FCL). However, all these fatigue-monitoring tools are based on the fatigue damage calculation, Miner’s Rule [9].

As one of the earliest emerging theories for fatigue damage calculation, Miner’s rule has been chiefly used in many western countries in their aircraft life monitoring practice. The rule can be expressed as Equation (1), where λ is the total life of the component, *n*_i_ is the number of cycles applied at a specific load amplitude, and *N*_i_ is the number of cycles to failure at that load amplitude [10]

(1) $$\lambda Ʃ\frac{n_{i}}{N_{i}}=Q$$

RMAF aircraft fleet consists of both Safe Life and basic Damage Tolerance fatigue design. The safe life concept is adopted on fighter aircraft where their fatigue life is monitored either by airframe hours or fatigue index. The Damage Tolerance concept is used for the transport aircraft, which has the inspection method’s safety. Fatigue failures in the aircraft structure are either induced by operational loading or cyclic loading [11].

## 4. Forms of Corrosion Occurring on Critical Aircraft Structure

**Figure 2:**
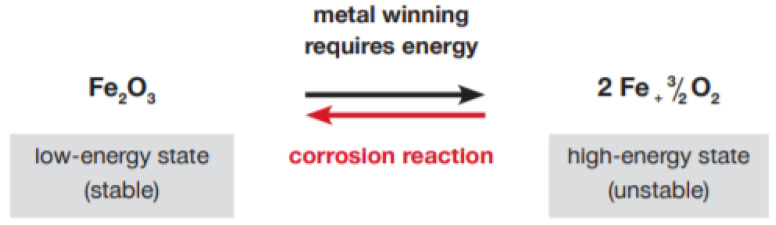
Chemical reactions of iron during corrosion and the metal-winning process [12].

Since many of the RMAF bases are located close to the seashore, these areas are the salt-laden areas prone to corrosion. Corrosion which is the interaction between the primary metal and its surrounding environment causes changes in the material’s properties. It either causes structural degradation, which is visible, or reduces the material’s structural life or performance. The most commonly used metals in the industry are carbon steel, stainless steel, zinc, copper, and aluminum. The primary metal corrosion equation is depicted in Figure 2.

Corrosion can be related to chemical reactions. This is because corrosion processes will occur when we have conditions suitable for the related chemical reaction. The key elements that are required in a corrosion reaction to take place are as below:

- Conducting Metal;
- Electrolyte (thin layer of moisture); and
- Oxygen for the cathodic reaction.

### 4.1. Forms of Corrosion

There are various corrosion types, such as uniform corrosion, pitting corrosion, crevice corrosion, environmental induced cracking, intergranular corrosion, and galvanic corrosion. However, based on the data collection and structural audit performed on the RMAF aircraft, the most common corrosion types are pitting and intergranular corrosion.

#### 4.1.1. Pitting Corrosion

**Figure 3:**
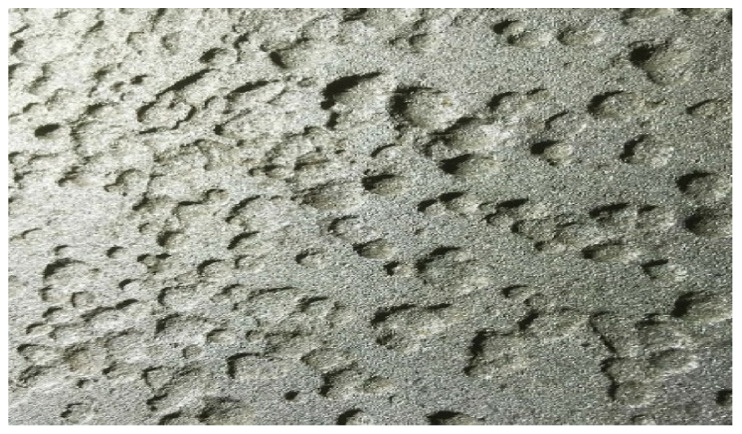
Example of Pitting Corrosion [12].

Pitting corrosion as shown in Figure 3 is a localized corrosion that causes small holes or pits on the metal surface. This type of corrosion occurs typically on passive metals such as aluminium, titanium, and stainless steel. This corrosion is initiated by a local breakdown of the passive layer. Pitting corrosion is also said to be problematic since it is not detectable in its early stages. It will be in the form of small pinholes on the surface. Its amount of material being removed by the reaction is generally unknown, making it more challenging to detect and predict. These corrosions can be the initiation point for the more critical corrosion type, such as stress corrosion cracking. 

#### 4.1.2. Intergranular Corrosion

**Figure 4:**
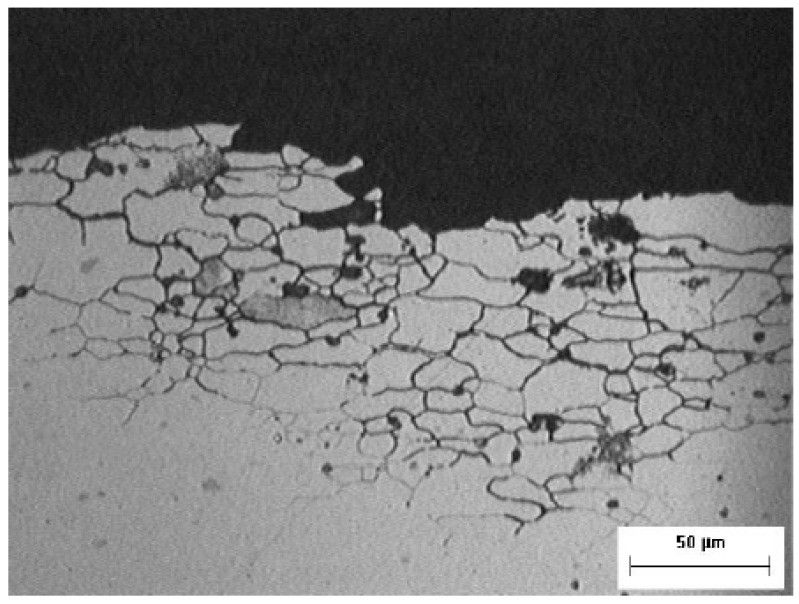
Example of Intergranular Corrosion [13].

Intergranular corrosion is a unique type of localized corrosion which is depicted in Figure 4. The corrosive attack occurs in a narrow path, mainly along the grain boundary of the metal structure. The most popular effect due to this type of corrosion is the rapid disintegration of the metal. Although there may be protection such as paint and primer on the metal, intergranular corrosion can occur below this surface if there is a minor scratch or damage to the protection layer.

## 5. RMAF Aircraft Primary Structure Corrosion Damage Review

Currently, the aviation industry is speedily developing with better materials that are more fatigue and corrosion-proof. However, this progress can be hindered by aggressive corrosion inducing environments [14]. The types of corrosion that occur on each type of aircraft may differ in various ways. Atmospheric corrosion is the most common type of corrosion that is affecting the RMAF aircraft structure. This occurs when there is an interaction between metal and the environment. The main issue with the corrosion damage in the RMAF fleet is that the occurrence is affected by many parameters such as geographical location, aircraft range, local climate, and weather change.

Furthermore, the aircraft in service stay on the ground at their respective bases either due to maintenance downtime or no operation flying. This is an important factor when considering the corrosive processes.

### 5.1. Trainer Aircraft

**Figure 5:**
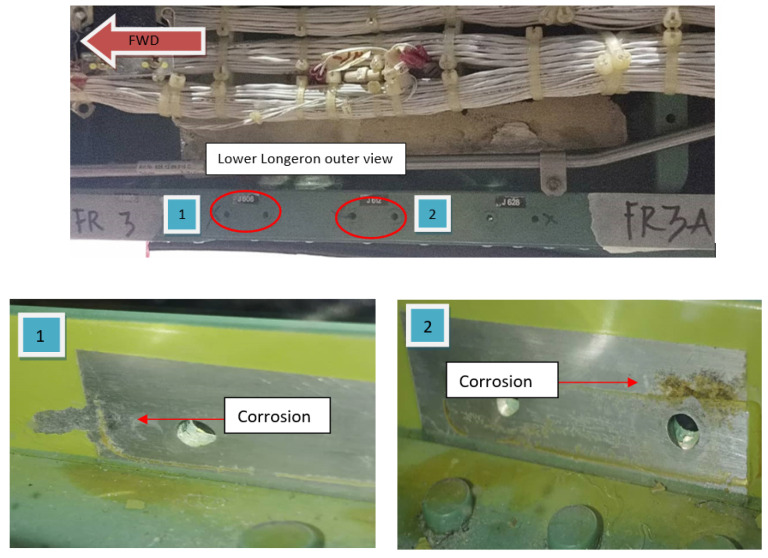
Intergranular corrosion on upper longeron.

**Figure 6:**
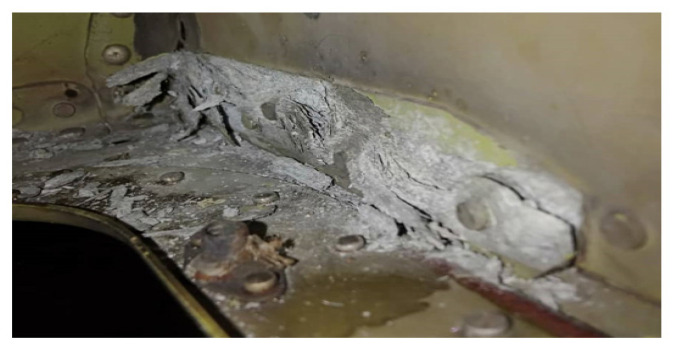
Corrosion on Wing Spar.

The RMAF is currently using the PC-7MkII aircraft as its trainer fleet. The PC-7MkII aircraft, a turboprop trainer type aircraft, is manufactured by Pilatus Aircraft Ltd. This aircraft is used for basic flying training or fighter conversion training and is intended for student pilots. Its airframe and avionics are similar to those of the PC-9M, which has a modular design [15]. Severe corrosion was found at the upper longeron, lower longeron, and wing spar when undergoing structural maintenance. These areas are listed as Primary Structure Element. The corrosion on the upper longeron is depicted in Figure 5 while the corrosion on the wing spar is shown in Figure 6.

### 5.2. Helicopter

**Figure 7:**
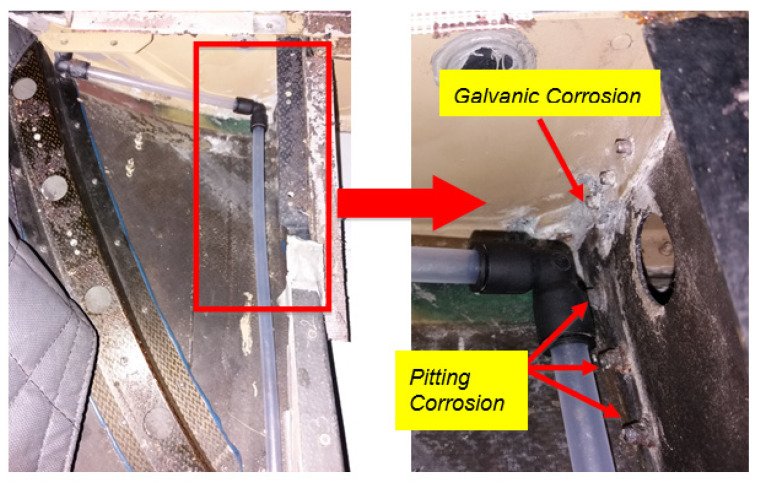
Corrosion at Frame X7225 till X7225.

The EC725AP is the helicopter that is serving the RMAF. These helicopters based at Kuantan and Labuan airbase serve as tactical and search and rescue aircraft. Most of their structure is built from composite, however, there are some parts manufactured from aluminium alloy. Based on the depot-level maintenance carried out on one of the aircraft, it was found that the area of Frame X7225 till X7725 as shown in Figure 7 had been affected by severe corrosion [16].

### 5.3. Transport

**Figure 8:**
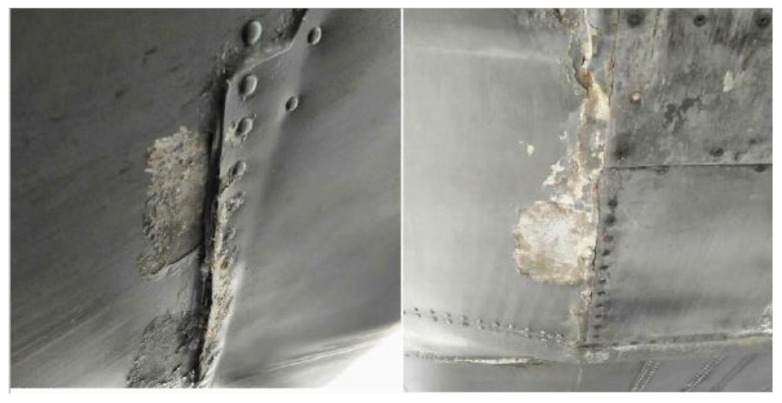
Corrosion at Lower Skin area.

The Lockheed C-130 Hercules, also known as C-130H, is a military transport aircraft operated with a four-engine turboprop. Produced by Lockheed Martin, the aircraft was designed for troop, medevac, and cargo transport. Furthermore, this aircraft has many more capabilities, such as cloud seeding and air-to-air refueling. With its multirole capabilities and robust operating condition, this aircraft is subjected to corrosion and fatigue damage. It is currently located at Subang and Labuan Air Base. Based on the findings during its primary structural inspection, corrosion was found at the ramp and lower skin area as shown in Figure 8.

### 5.4. Microscopic Examination and Fractographic Examination

**Figure 9:**
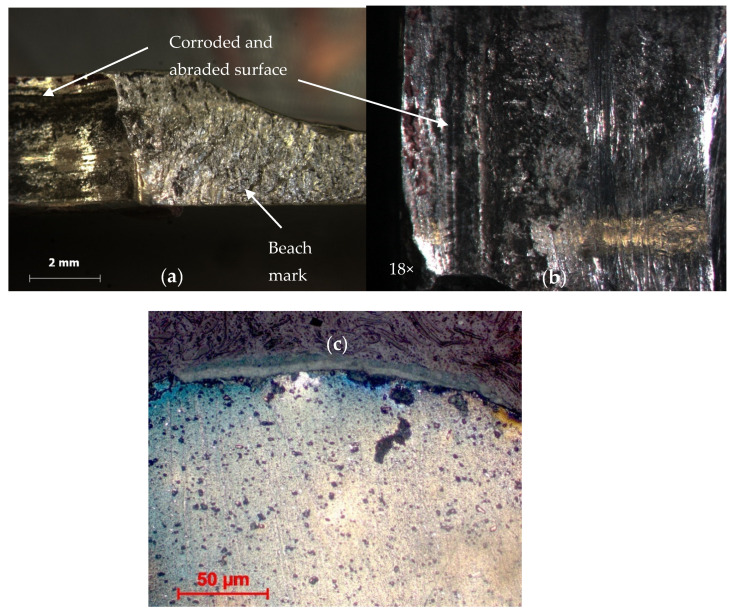
Fracture surface due to corrosion and fatigue. (**a**) Beach mark and corrosion of frame X7225 of EC725. (**b**) Close up image (18×) on the specimen. (**c**) Close up image of C130H lower skin area.

Examination at low magnification using an optical stereo microscope on the fracture surface on most of the structure revealed a beach mark, as shown in Figure 9a. A closed-up view on the surface found it was severely corroded and had abraded, as shown in Figure 9b.

**Figure 10:**
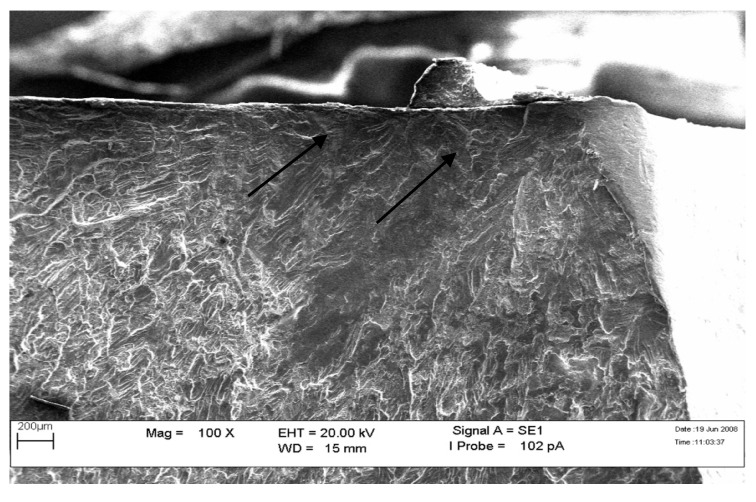
SEM image; arrow shows the fatigue striation (100×).

**Figure 11:**
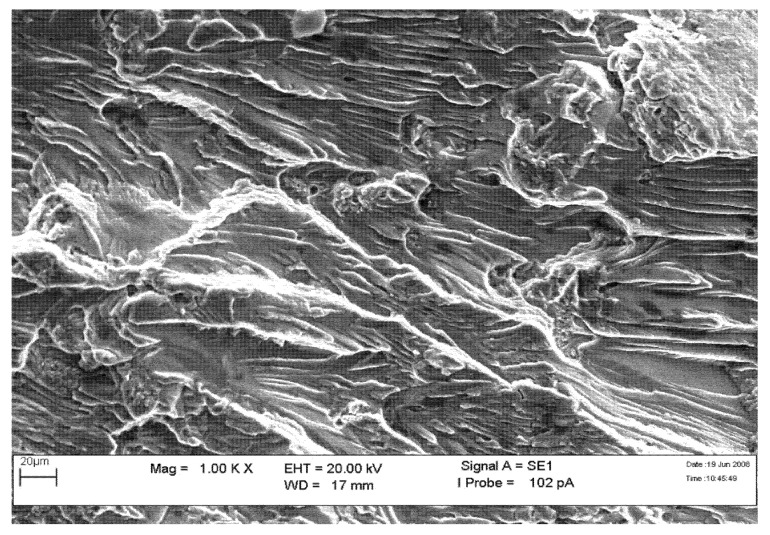
SEM image shows the fatigue striation (1000×).

The fracture surface’s crack initiation and beach mark region were examined using Scanning Electron Microscope (SEM) (Zeiss, Oberkochen, Germany). The presence of fatigue striation shown in Figure 10 and Figure 11 indicates that the primary structure assembly had failed due to fatigue failure.

## 6. Structural Integrity Evaluation

Based on the current structural corrosion condition resulting in fatigue, a structural integrity evaluation was proposed to be carried out on the fleet. The RMAF uses the PC-7 Mk II trainer aircraft for basic pilot training. There are currently 21 aircraft in the fleet, which was received in three batches between the year 2000 and 2016. During the 300 h/1 year structural inspection, it was identified that the upper longeron had issues of corrosion that could affect the structural integrity of the aircraft.

Based on RMAF [17], there were a total of 16 aircraft which were affected by this corrosion issue. Hence a total of five aircraft were chosen for further analysis on corrosion severity level. Since there was one out of the five aircraft with minimum corrosion, the RMAF top echelon decided that that aircraft would be chosen as the sample for the structural investigation and analysis to determine whether it was suitable for further flying. The aircraft usage data (flying hours, on-ground hours, and crack mapping) were also gathered for all the affected airframes. These data recorded after every flight are critical data to provide the basis for future alteration of the maintenance plan.

### 6.1. Methodology

#### 6.1.1. Strength Reduction Analysis

The corrosion that was spotted on the upper and lower longeron blended out of the corroded surface, which resulted in thickness reduction. Consequently, there was a need to carry out a stress analysis on the effect of reduced thickness against the strength of the longeron using Finite Element Analysis (FEA) [18].

**Figure 12:**
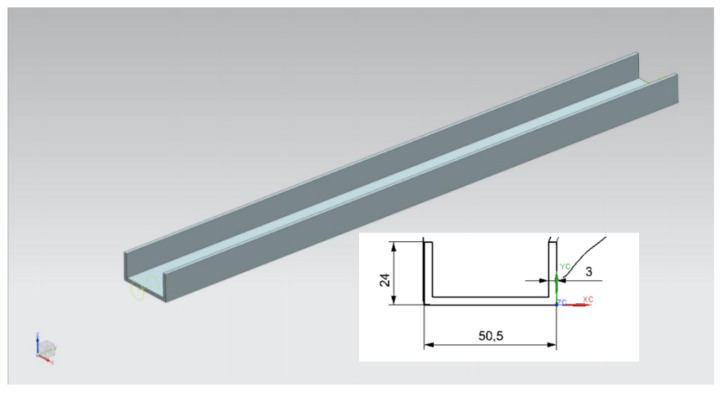
CAD model of the C-section of the longeron.

In this analysis, the FEA will be performed on the upper longeron structure modelled as a C-beam structure model. A 3D scanning was conducted on the actual longeron of the aircraft. This was followed by a CAD modelling of the C-sections of the longeron beam structure as shown in Figure 12. An FEA meshing and its boundary conditions were designated on the longeron 3D model. Finally, the strength reduction analysis against the thickness reduction of the longeron was carried out. The input parameters for the upper longeron design are shown in Table 1.

Boundary conditions were applied at 1/3 of the total length of the CAD model. Two fixed boundary conditions were placed on the FEA model to imitate the landing gear support location on the aircraft’s longeron.

**Figure 13:**
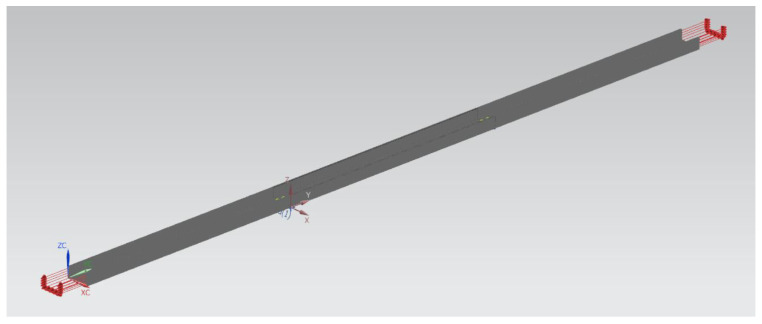
Load applied at the longeron.

An applied loading was placed on both ends of the longeron, as shown in Figure 13. Loading was altered by varying its value until the stress experienced by the longeron approaching its yield stress. The yield strength value of aluminium alloy Al2024 is 289.579 Mpa.

#### 6.1.2. Corrosion Blend Out and Strain Gauge Installation

Regarding the FEA simulation, the affected aircraft longeron was equipped with several strain gauges to record the flight test’s strain sequence. The FEA method was developed with the experimental activity and now represents a valuable tool in the numerical evaluation of local stress and strain. However, the results obtained will be validated using the flight test, which will record the fatigue strength using the strain gauge [19].

**Figure 14:**
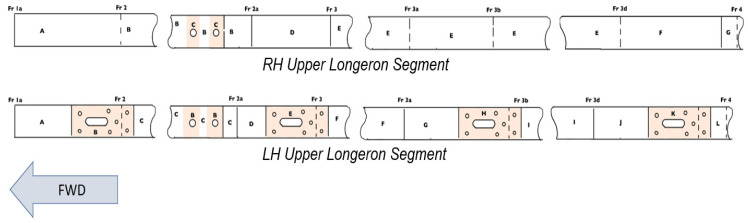
Segments on the longeron for inspection.

**Figure 15:**
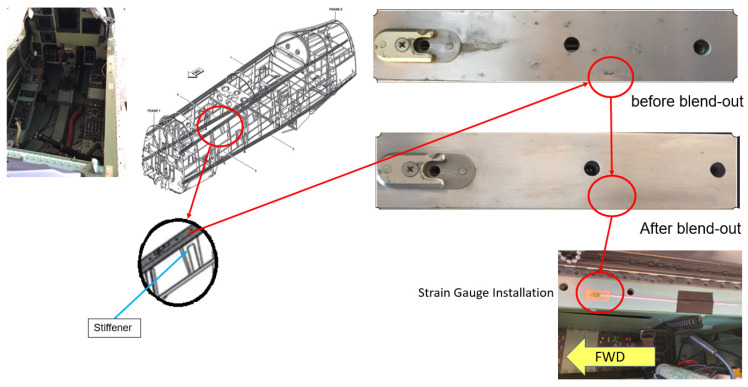
Blend out of corrosion and strain gauge installation.

The left-hand (LH) and right-hand (RH) longeron were segmented based on Figure 14 for corrosion inspection. Upon blend of the corrosion, a strain gauge was installed at Section C on the RH longeron and Section B on the LH longeron as shown in Figure 15.

##### Longeron Thickness Assessment

The longeron thickness was assessed using the Non-Destructive Test (NDT). The method used was Liquid Penetrant (LPI) and Ultrasonic Test (UT). The level of corrosion was stated as below:

- Light < 0.0254 mm;
- 0.0254 mm < Moderate < 0.254 mm; and
- Severe > 0.254 mm.

**Figure 16:**
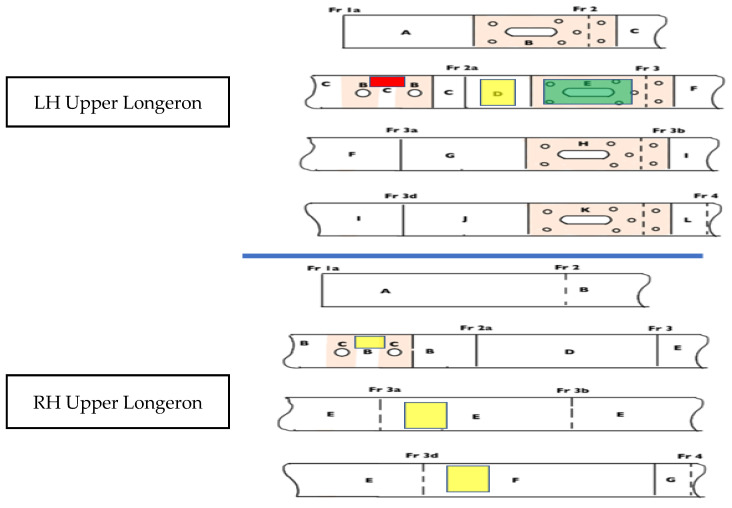
Inspection result for the RH and LH upper longeron.

There were a few corrosion spots on the LH and RH longeron. Corrosion treatment work was carried out, which in return reduced the thickness of the longeron. The longeron has a thickness of 2.7 +/− 0.2 mm. Considering that the longeron’s initial thickness (datum) is 2.7 mm, the structural area was treated and segmentized. Based on Table 2 and Figure 16, there was a thickness loss of 0.36 mm on the LH longeron. It is located between the two-lifting point. The detailed inspection and treatment result is depicted in Table 2.

#### 6.1.3. Data Gathering Process

Stress measurement is the amount of load or strength which is acting on the longeron under operation. The amount of stress that the longeron can withstand is known as its yield strength or safety factor. Besides that, it is also crucial to determine the amount of stress acting longer to produce its optimum design and the assurance of strength and safety. The strain gauge is used to detect the strain that acts on the affected longeron during flight tests. The strain is known as the deformation’s quantity—elongation or contraction of material in proportion to the applied external force.

**Figure 17:**
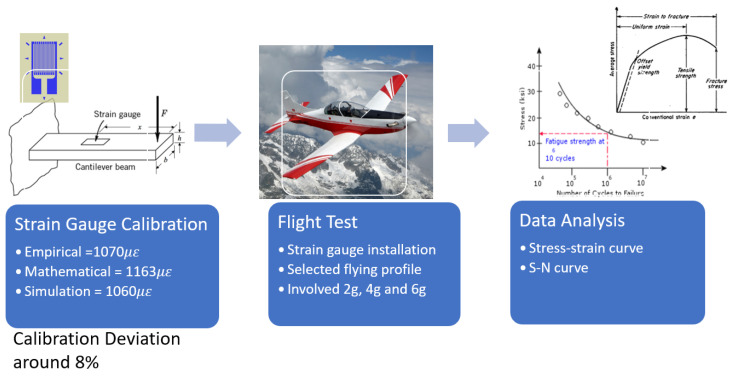
Data Gathering Process

Before installation, the strain gauge was calibrated to ensure its required, resulting output. The theory of simple bending is used to determine the location for the strain gauge installation. When acting any load on the beam or any force on its axis, the beam is subjected to deformation. The bending is also known as axial deformation. The deformation is due to the shear force and bending moment which is acting on it. Based on Figure 17, the deviation for calibrating using empirical, mathematical, and simulation is around 8%. The deviation value is acceptable since our limit is set at a maximum of 15%.

The aircraft was flown on a series of flight tests using various G-load profiles (2g, 4g, 6g, and 7g). The flight test was carried out to evaluate the corrosion-affected longeron’s structural strength and assess the longeron’s stress level. The ideal reference that can be used to determine the maximum stress against the number of cycles of failure for any material before it reaches its failure point will be the S-N curve [20]. Using the maximum stresses value at different g conditions, a corresponding number of cycles to failure is obtained from the S-N curve of aluminium 2024 [21].

## 7. Result and Discussion

### 7.1. FEA Simulation Result on Upper Longeron

**Figure 18:**
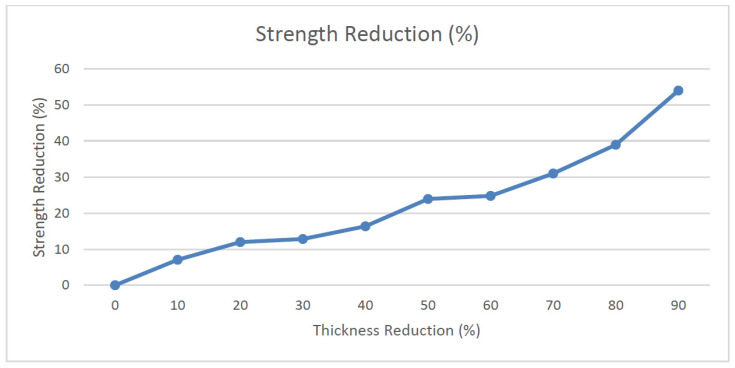
Strength reduction vs. Thickness reduction for upper longeron

Figure 18 below depicts the graph of strength reduction in percentage versus thickness reduction with an increment of 0.3 mm. Table 3 shows the details of the strength reduction of the upper longeron.

Based on Md Fuad Shah Koslan and Bakar [22], the area with the highest corrosion severity is Area B. The minimum remaining thickness is approximately around 2.34 mm with a loss of 0.36 mm thickness. Referring to Table 3, it is evident that with a remaining thickness of 2.34 mm, the maximum load allowable at that area is around 9950 N before the object fails. The amount of stress obtained in the area is 35.58 Mpa. The strength reduction is approximately around 11.94%.

#### Flight Test Data

The flight test was carried out to obtain the strain data from the strain gauge installed on the longerons. The flight profile used in this sortie was a basic flying maneuver with acceleration at specific intervals. The sensors are mainly subjected to compressive, bending, or torsional loads during operation due to the design assumptions. In this case, there is no risk of fatigue or static damage by direct tearing of the monitored element. However, these records provide indirect information about the overall load level of the structure.

**Figure 19:**
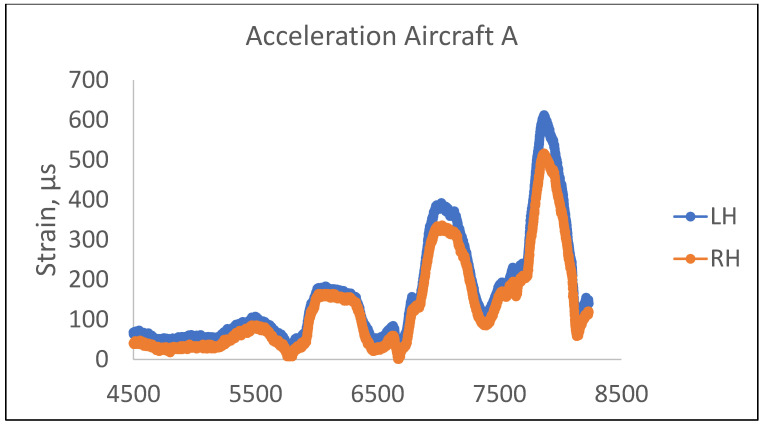
Acceleration data from aircraft flight test.

Based on Figure 19 and Table 4, there is a variation of strain recorded on either side of the longeron. The LH longeron has the highest strain recorded during the 6g acceleration, approximately around 612.5 με (44.1 Mpa). Based on the corrosion severity data in Table 2, the LH longeron recorded the lowest remaining thickness, which contributed to the stress concentration point and recorded the highest strain. This also most likely shows that the location is subjected to a reduction in fatigue strength.

### 7.2. Stress Concentration Factor

Designing aircraft structures or analyzing structures that are having fatigue failures is done based on sample specimens. These specimens will be either damage-induced, salt-sprayed, or continuous cyclic loaded to determine their life. The main assumption that can be made is that any material’s static strength is usually affected by notches, holes, and fillets. Besides that, the areas that have this kind of dimension will be the stress-concentrated area. The stress-concentration factor Kc is the area test stress ratio in the notch region (or other stress concentrators) to the corresponding nominal stress [23].

**Figure 20:**
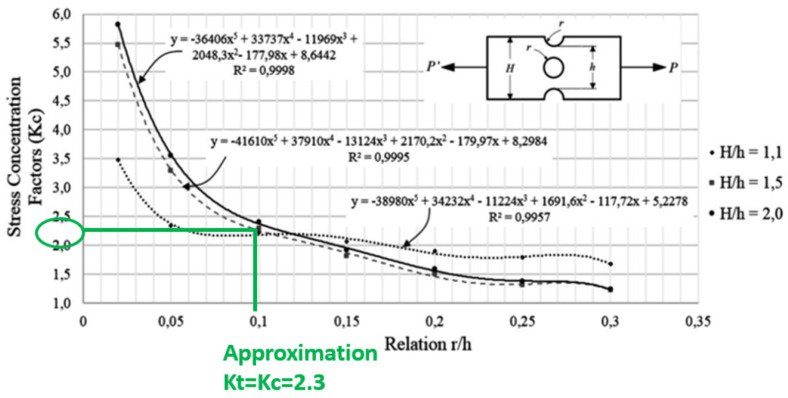
Stress concentration factor for notch Aluminum specimen [25].

In the combined discontinuity central hole and groove, it is observed that the concentration factor decreases as the radius of the discontinuities (hole and groove) increase, consistent with the behavior exhibited in most concentration factors with simple discontinuities. Additionally, it can be concluded that there is no definite trend of this parameter concerning the relation between widths (H/h) of the flat plate, which is due to the maximum value of stress placed in some cases in the central hole and others in the groove [24]. The graph (Figure 20) equations are the governing equation for the points on the graph line for the relation between r/h and stress concentration factor.

Based on the graph in Figure 20, assuming the longeron geometry is a rectangular bar with a notch and transverse hole, to obtain the stress concentration factor Kc, the relation between r/h is in equation 2. Hence the Kc is approximately 2.3 ≈ 2.0, which is depicted by equation H/h = 2.0.

(2) $$Relation=\frac{r}{h}=\frac{4.88}{50}=0.0976$$

### 7.3. Fatigue Life Cycle

**Figure 21:**
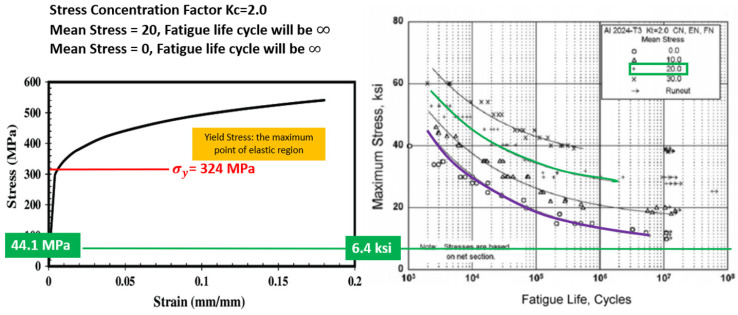
Equivalent Stress and Fatigue Life Cycle [26].

Based on Figure 21, the stress–strain graph depicts the true stress–strain behavior of the AA2024-T3 alloy. This graph, which is also referenced in the Metallic Materials Properties Development and Standardization (MMPDS), states the maximum yield stress for the AA2024. Taking into consideration that Kc is 2.0, the mean stress is calculated as below.

$$\sigma_{mean}=\frac{\sigma_{\max at\;6g}-\sigma_{\min at\;v=0}}{2}\;=\frac{44-0}{2}\;=22\;\left(\approx \;20\right)$$

With the Kc value of 2.0 (Figure 20), the mean stress curve chosen is 20 based on the result above. Based on the evaluation, the affected longeron’s maximum stress during 6g loading on the upper longeron was 44.1 MPa (Table 4). The maximum yield stress for AA2024 is 324 Mpa based on the stress–strain graph (Figure 21). The longeron is safe because the flight test’s stress magnitude is less than the structural material’s yield strength. Figure 21 also depicts the typical S-N diagram for fatigue behavior for AA2024. Therefore, at 44.1 MPa, which is approximately 6.4 ksi and with mean stress of 20, the fatigue life cycle is ∞.

## 8. Conclusions

Stress simulation analysis was performed on the upper longeron structure with its maximum allowable stress determined based on the thickness reduction. With a 50% decrease in thickness, it will result in more than 25% reduction in maximum loading for the longeron. It was found that, based on the flight test, the maximum yield strength obtained at the highest G load was 44.10 MPa. This value is much lower than the maximum yield strength of the AA2024 material which is 324 MPa with a safety factor of 7.3. Comparing the result from the simulation and flight test, the structural integrity of the longeron is within the operating limits of the aircraft.

Usually, the fatigue crack initiates in a structure where the maximum tensile stress is located. The fatigue calculation is carried out for the prediction of the structural life of the longeron structure. The highest thickness loss at the longeron is approximately 13%. The stress concentrated on the most severe area is still within the allowable limitation based on the flight test and simulation carried out. However, the maximum allowable flight operating envelope will be at 6G.

The periodic maintenance of the longeron structure is around 300 hrs. However, currently corrosion is found to be present even before the planned maintenance interval. The proposed maintenance interval will be performed at a shorter interval to analyse the structural condition to prevent corrosion. Table 5 depicts the proposed maintenance interval. The method of inspection used will be the Non-Destructive Test (Liquid Penetrant and Eddy Current).

An Aircraft Structure Integrity Program should also be established for this particular fleet to monitor its structure integrity [27]. The four critical elements of ASIP: Usage Monitoring, Condition Monitoring, Fatigue Management, and Environmental Degradation Management should be planned and performed to collect essential data. This data is very crucial for future structure life assessment and service life extension programs. Data recorded during operation will be gathered and stored, allowing automated estimation of possible damage and scheduling necessary for inspections and repairs.

## Figures and Tables

**Table 1:** Details of the longerons dimension.

No	Detail	Measurement (mm)
1	Length	1536.7
2	Width	50.5
3	Height	24
4	Thickness	3
5	Area	279.6 mm^2^

**Table 2:** Inspection result for RH and LH upper longeron.

**LH Upper Longeron**				
**Section**	**Area** **Refer**	**Minimum Remaining Thickness (mm**)	**Thickness lost (mm)**	**Severity Level**
1a-2 (except Area B)	A	2.7	-	-
Within 10 mm of chafing plate and 10 mm of hoisting point holes	B	2.34	0.36	Severe
2-2a (except Area B)	C	2.7	-	-
2a-3 (except Area E)	D	2.67	0.03	Moderate
Within 10 mm of chafing plate	E	2.69	0.02	Light
3-3a (except Area E)	F	2.7	-	-
3a-3b (except Area I)	G	2.7	-	-
Within 10 mm of chafing plate	H	2.7	-	-
3b-3d	I	2.7	-	-
3d-10 mm forward of chafing plate	J	2.7	-	-
Within 10 mm of chafing plate	K	2.7	-	-
Within 10 mm aft of chafing plate-aft edge of the shear panel	L	2.7	-	-
**RH Upper Longeron**				
**Section**	**Area** **Refer**	**Minimum Remaining Thickness**	**Thickness lost (mm)**	**Severity Level**
1a-2	A	2.7	-	-
2-2a (except Area C)	B	2.7	-	-
Within 10 mm of hoisting point	C	2.63	0.07	moderate
2a-3	D	2.7	-	-
3-3d	E	2.63	0.07	moderate
3d-10 mm forward of frame 4	F	2.64	0.06	moderate
Frame 4- aft edge of the shear panel	G	2.7	-	-

**Table 3:** Details of analysis based on the remaining thickness.

Thickness (mm)	Max Load (N)	Strength Reduction (%)	Stress (Mpa)	Thickness Reduction (%)
2.7	10,500	0	37.55	0
2.4	9950	11.94690265	35.58	20
2.1	9850	12.83185841	35.22	30
1.8	9450	16.37168142	33.79	40
1.5	8600	23.89380531	30.75	50
1.2	8500	24.77876106	30.40	60
0.9	7800	30.97345133	27.89	70
0.6	6900	38.9380531	24.67	80
0.3	5200	53.98230088	18.59	90

**Table 4:** Strain and stress data.

	Date	Time	Max Strain at 2g (με)		Max Strain at 4g (με)		Max Strain at 6g (με)				
			LH	RH	LH	RH	LH	RH	Max Strain (με)	Max Stress (MPa)	SF= $\frac{\sigma_{yield}}{\sigma_{actual}}$
Aircraft A	26 February 2020	0730 H	183.3	164.7	392.3	336.2	612.5	516.4	612.5	44.100 (6g on LH)	7.3

**Table 5:** New proposed maintenance interval.

Interval			
1	2	3	4
**75 h** Inspection task: Visual Inspection and surface protection removal Eddy Current and Liquid Penetrant Inspection	**150 h** Inspection task: Visual Inspection and surface protection removal Eddy Current and Liquid Penetrant Inspection	**225 h** Inspection task: Visual Inspection and surface protection removal Eddy Current and Liquid Penetrant Inspection	**300 h** Inspection task: Visual Inspection and surface protection removal Eddy Current and Liquid Penetrant Inspection

## Data Availability

The data presented in this study are available on request from the corresponding author.
